# An Entertaining Periodical

**Published:** 1889-06

**Authors:** 


AFTER the moral and religious education of the family, we know of nothing that will confer a more lasting and permanent benefit than that most instructive and, at the same time, entertaining, periodical, the *Scientific American.* It should be a visitor in every home, where its work will be found not to be idle. It is of special value to the machinist, the engineer, and the mechanic, but it is of equal value to the farming and mercantile community, and to all who are of an inventive or ingenious turn of mind. It will be found invaluable to those, whether young or old, who are fond of using tools as a recreation. The subscription price is only $1.00 for four months, or $3.00 a year. The publishers are the old-established house of Munn & Co., 361 Broadway, N. Y.

